# Acceleration of tissue phase mapping by k-t BLAST: a detailed analysis of the influence of k-t-BLAST for the quantification of myocardial motion at 3T

**DOI:** 10.1186/1532-429X-13-5

**Published:** 2011-01-11

**Authors:** Anja Lutz, Axel Bornstedt, Robert Manzke, Patrick Etyngier, G Ulrich Nienhaus, Volker Rasche

**Affiliations:** 1 Department of Internal Medicine II, University Hospital of Ulm, Ulm, Baden-Württemberg, Germany; 2 Philips Research Europe, Hamburg, Germany; 3 Medisys Research Lab, Philips Healthcare, Suresnes, France; 4 Institute of Applied Physics Karlsruhe, Institute of Technology (KIT), Karlsruhe, Germany

## Abstract

**Background:**

The assessment of myocardial motion with tissue phase mapping (TPM) provides high spatiotemporal resolution and quantitative motion information in three directions. Today, whole volume coverage of the heart by TPM encoding at high spatial and temporal resolution is limited by long data acquisition times. Therefore, a significant increase in imaging speed without deterioration of the quantitative motion information is required. For this purpose, the k-t BLAST acceleration technique was combined with TPM black-blood functional imaging of the heart. Different k-t factors were evaluated with respect to their impact on the quantitative assessment of cardiac motion.

**Results:**

It is demonstrated that a k-t BLAST factor of two can be used with a marginal, but statistically significant deterioration of the quantitative motion data. Further increasing the k-t acceleration causes substantial alteration of the peak velocities and the motion pattern, but the temporal behavior of the contraction is well maintained up to an acceleration factor of six.

**Conclusions:**

The application of k-t BLAST for the acceleration of TPM appears feasible. A reduction of the acquisition time of almost 45% could be achieved without substantial loss of quantitative motion information.

## Background

Quantification of myocardial mechanics is supposed to provide an improved understanding of cardiac motion as well as to enable a more detailed assessment of certain myocardial diseases such as cardiac insufficiency. A major limitation in quantification of cardiac function is the long measurement time required for three-dimensional (3D) velocity encoded imaging. However, in diagnosis and staging of certain cardiac diseases and for therapy selection, 3D functional information of the myocardial motion appears mandatory. Especially for the selection of patients eligible for cardiac resynchronization therapy (CRT), quantification of the 3D-cardiac motion appears paramount to reduce or completely avoid non-responders, which represent 30% of treated patients using current selection criteria [1].

Four main approaches have been introduced for the assessment of myocardial mechanics including tagging [2-4], displacement encoding with stimulated echoes (DENSE) [5-8], strain encoding (SENC) [9] and tissue phase mapping (TPM) [10-14], which has also been introduced as phase contrast velocity encoded imaging [15,16] of tissue.

In the tagging technique, lines or a grid are mapped onto the myocardium by either spatial modulation techniques [2,3] or a DANTE pulse train in the presence of a frequency-encoding gradient [17]. Direct analysis of the tag-deformation over the cardiac cycle provides access to the inter-voxel strain and velocity of the myocardium, but is limited by the spatial resolution of the tag pattern. This can partly be solved by applying dedicated post-processing techniques such as the harmonic phase approach (HARP) [18].

The DENSE approach directly encodes displacements over long time intervals at high spatial density [5]. Due to the long displacement encoding intervals, data acquisition is very slow.

In the SENC technique, an intra-voxel tag-pattern is used for the assessment of the intra-voxel strain, which enables the assessment of the stiffness of the myocardium. The application of the SENC technique as the sole technique for the assessment of the cardiac function is limited by the lack of information on the inter-voxel strain and myocardial velocities.

In TPM, the myocardial velocity is directly encoded by the application of bipolar gradients causing the spins to acquire a phase that is directly proportional to their velocity. Since the direction of the velocity encoding gradients can be chosen freely, TPM enables the quantitative assessment of the 3D flow vector. Wide application of TPM is still limited by the long acquisition times, which preclude large volume coverage at sufficient spatial resolution and may introduce image deterioration due to varying respiratory or irregular cardiac motion [19].

For acceleration of the image acquisition, several methods have been introduced. Local imaging techniques aim at reducing the field-of-view (FOV) to a confined area containing the heart [19-21]. Its sensitivity to patient motion and the required complicated planning of the anatomy have limited their clinical application. More promising techniques employ correlations in k-space or image space like sensitivity encoding (SENSE) [22], generalized autocalibrating partially parallel acquisitions (GRAPPA) [23] and partial Fourier methods [24].

View sharing exploits temporal correlations by reusing some of the same k-space data in order to reconstruct additional images [25-28]. With view sharing, a decrease of the acquisition time of 37.5% could be obtained without significant deterioration of the velocity mapping data [28]. Temporal correlations are also exploited in the UNFOLD approach (unaliasing by Fourier-encoding the overlaps using temporal dimension) [29,30], which avoids aliasing resulting from undersampling by shifting the sampling function in time, such that Fourier transformation through time can resolve these overlaps.

More recently dedicated techniques like k-t BLAST and k-t SENSE exploiting both correlations in k-space and in time by sparse sampling have been introduced [31-34]. The resulting aliased images in the reciprocal spatio-temporal frequency domain are resolved using the information of prior acquired low resolution data leading to aliasing free images. It seems feasible to use k-t BLAST for accelerated image data acquisition in applications involving quasiperiodic motion such as the heart.

K-t BLAST has been applied to various applications in the medical field [35-39]. Especially it has proven its applicability to velocity encoded imaging for flow quantification [32,40-42].

The objective of this contribution is to investigate the potential of k-t BLAST for the acceleration of TPM image acquisition at 3 T. The k-t BLAST technique was evaluated applying different acceleration factors for the assessment of quantitative myocardial motion and compared to the non accelerated technique.

## Methods

### Data acquisition

20 adult volunteers (7 females, 13 males, age 29 ± 11 years) were enrolled in the study [kt-group]. All volunteers enrolled underwent one non-k-t BLAST TPM data acquisition and 6 k-t BLAST accelerated sequences with k-t BLAST factors R ranging from 2 to 7. The sequence order was randomized to reduce the influence of physiological variations on the myocardial motion between the acquired sequences. To assess of the reproducibility of the approach, the non k-t BLAST protocol (no-kt) was repeated twice in 20 additional volunteers (fourteen males, six females, age 28 ± 6 years) [reference group]. The study protocols were approved by the local ethics committee and informed written consent was obtained from all volunteers prior to the MRI examination.

All MRI scans were performed on a 3 T whole body MR scanner (Achieva 3.0 T, Philips, Best, The Netherlands) with a 32 [2 × 4 × 4] channel phased array cardiac coil. A vector ECG was applied for cardiac triggering. Breath-hold cine cardiac two-chamber and four-chamber views were acquired to define the short axis image geometry, which was used in all subsequent acquisitions.

The TPM acquisition was performed applying a respiratory navigated segmented and velocity encoded cardiac triggered gradient echo sequence. The acquisition parameters were as follows: TE/TR = 4.7 ms/7.1 ms, flip angle α = 15°, FOV = 340 × 340 mm^2^, acquisition matrix (M*P) = 172*168, slice thickness = 8 mm, in plane resolution: 2 × 2 mm^2^, 3 k-lines per segment and one startup echo, VENC = 30 cm/s in all 3 encoding directions. The acquisition window was 90% of the RR interval. The duration of the saturation module consisting of saturation pulses and spoiler gradients was 12 ms. The phase interval without k-t BLAST acceleration was 40.4 ms. The navigator was 15.5 ms long, the navigator feedback time 5 ms. To solely investigate the impact of the k-t BLAST, data acquisition was not combined with further acceleration techniques like view sharing or parallel imaging. The TPM encoding was performed in all 3 spatial orientations in consecutive heart beats. A conventional pencil beam navigator through the dome of the right hemi-diaphragm was applied at each start of the cardiac cycle for respiratory gating [43,44].

To avoid flow related artifacts in the phase images caused by the strong blood flow in the ventricle and to improve the delineation of myocardium and blood, two saturation pulses were incorporated superior and inferior of the imaged slice to generate black-blood contrast [13]. To avoid idle times due to high SAR demands of the sequence at 3 T, the saturation pulses were applied alternating and the maximal B1-amplitude of the RF pulses was optimized to 8 μT [45].

For the non-accelerated technique and a heart rate of 60 beats per minute, the maximum number of heart phases was 21 and the nominal scan duration was 225 s. In this study, the number of acquired cardiac phases was a multiple integer of the k-t BLAST factor to ensure similar undersampling factors in all regions of k-space. The respective nominal scan times and maximum number of acquired phases for different acceleration factors are listed in table 1 exemplary for a heart rate of 60 beats per minute. For all k-t BLAST measurements, the number of k_y _phase encoding steps for the training scan was set to eleven. Please note, that the acceleration factor only impacts the acquisition stage, whereas the length of the training stage remains constant. Hence the relative gain in acquisition speed decreases with increasing k-t factors.

**Table 1:** Relationship between k-t BLAST factor, maximum number of heart phases and scan time.

K-t BLAST acceleration factor	Maximal heart phases	Scan time
no	21	225
2	20	125
3	21	85
4	20	69
5	20	57
6	18	49
7	21	45

Relationship between k-t BLAST factor, maximum number of heart phases and scan time for a heart rate of 60 beats per minute

### Data analysis

The TPM MR images were processed by in house developed MATLAB programs (MATLAB R2008a; Mathworks, Natick, Mass). The segmentation of the myocardium was performed automatically, relying on active contour techniques by incorporating a shape model. After the segmentation of the first phase, the information was propagated through the entire sequence by tracking profile intensities [46,47]. Background phase error correction was performed using a linear fit to the phase of static tissue as suggested earlier [48]. The radial (towards the center of the blood pool) and longitudinal (towards the apex of the heart) velocity curves were calculated from the acquired three-directional velocity vector. Prior to the analysis the velocity data were interpolated over time by cubic splines to provide a continuous velocity profile thus enabling the comparison of sequences with a different number of sampled heart phases. Physiologically, the accumulated phase over the entire heart cycle must result to zero. To compensate for non-linear phase error contributions, in a subsequent correction step the resulting velocity curves were shifted accordingly to meet the physiological conditions.

To assess the impact of the k-t factor on the quantitative velocity information the following parameters were derived:

• The systolic and diastolic peak velocities v_p,sys _and v_p,dias _were determined and the resulting velocity difference Δv = v_p,sys _- v_p,dias _was calculated for each sequence. Bland-Altman analysis was performed for the velocity differences Δv and the differences between Δv with and without kt-BLAST is denoted as Δv Diff. The peak factor PF was defined as the ratio of the velocity differences with and without k-t acceleration (kt-group) and accordingly as the ratio between the two reproducibility measurements (reference group): PF (seq. 1, seq.2) = Δv_seq.2_/Δv_seq.1_. This parameter was determined to evaluate whether k-t acceleration has an impact on clinical main features of the motion curve. Ideally, PF should be one. Due to the temporal smoothing of the k-t BLAST algorithm, it is expected that especially sharp peaks will be abraded. The PF for the radial and longitudinal velocity curves were referred as PF_r _and PF_l_.

• The normalized root mean square deviation nRMSD between the velocity curves with and without k-t acceleration (kt-group) and accordingly between the curves obtained by the reproducibility measurements (reference group) was calculated. The normalization was performed by dividing the root mean square deviation by Δv_nokt_. The radial and longitudinal nRMSDs were denoted as nRMSD_r _and nRMSD_l_.

• The correlation coefficients were determined for both groups to evaluate the statistical dependency between the velocity curves. Let u be the velocity of the first sequence to compare, and w be the velocity of the second sequence. Than u_i _and w_i _are the spline interpolated data at different time steps (step size: 0.01 ms), n is the number of time steps, ū and $\bar{\text{w}}$ are the mean values of u_i _and w_i _and σ_u _and σ_w _the corresponding standard deviation. The correlation coefficient c is than calculated as:$\text{c}=\overset{\text{n}}{\underset{\text{i}=1}{\Sigma }}\left[\left(\frac{{\text{u}}_{\text{i}}-\bar{\text{u}}}{\sigma_{\text{u}}}\right)\left(\frac{{\text{w}}_{\text{i}}-\bar{\text{w}}}{\sigma_{\text{w}}}\right)\right]$. Ideally the correlation coefficient should be one. The correlation coefficient between the radial velocity curves was denoted as c_r_, the correlation coefficient between the longitudinal velocity curves as c_l_.

• The times to the peak diastolic velocity of the radial and longitudinal velocity t_r,dias _and t_l,dias _was identified for each acquisition technique in order to determine, whether the temporal behavior of the myocardium is preserved for different acceleration factors. The temporal behavior is of special interest in various cardiac diseases such as cardiac asynchrony. Bland-Altman analysis was performed for t_r,dias _and t_l,dias _and the mean difference times Δt_r,dias _(seq.1, seq.2) = t_r,dias,seq.2_- t_r,dias,seq.1 _and Δt_l,dias _(seq.1, seq.2) = t_l,dias,seq.2_- t_l,dias,seq.1 _and their standard deviations over all volunteers were compared.

For the evaluation of significances an unpaired two-tailed student's t-test was performed. Values below 0.05 were considered to be significant. The variance of both reference and kt-group was considered to be the same.

## Results

**Figure 1:**
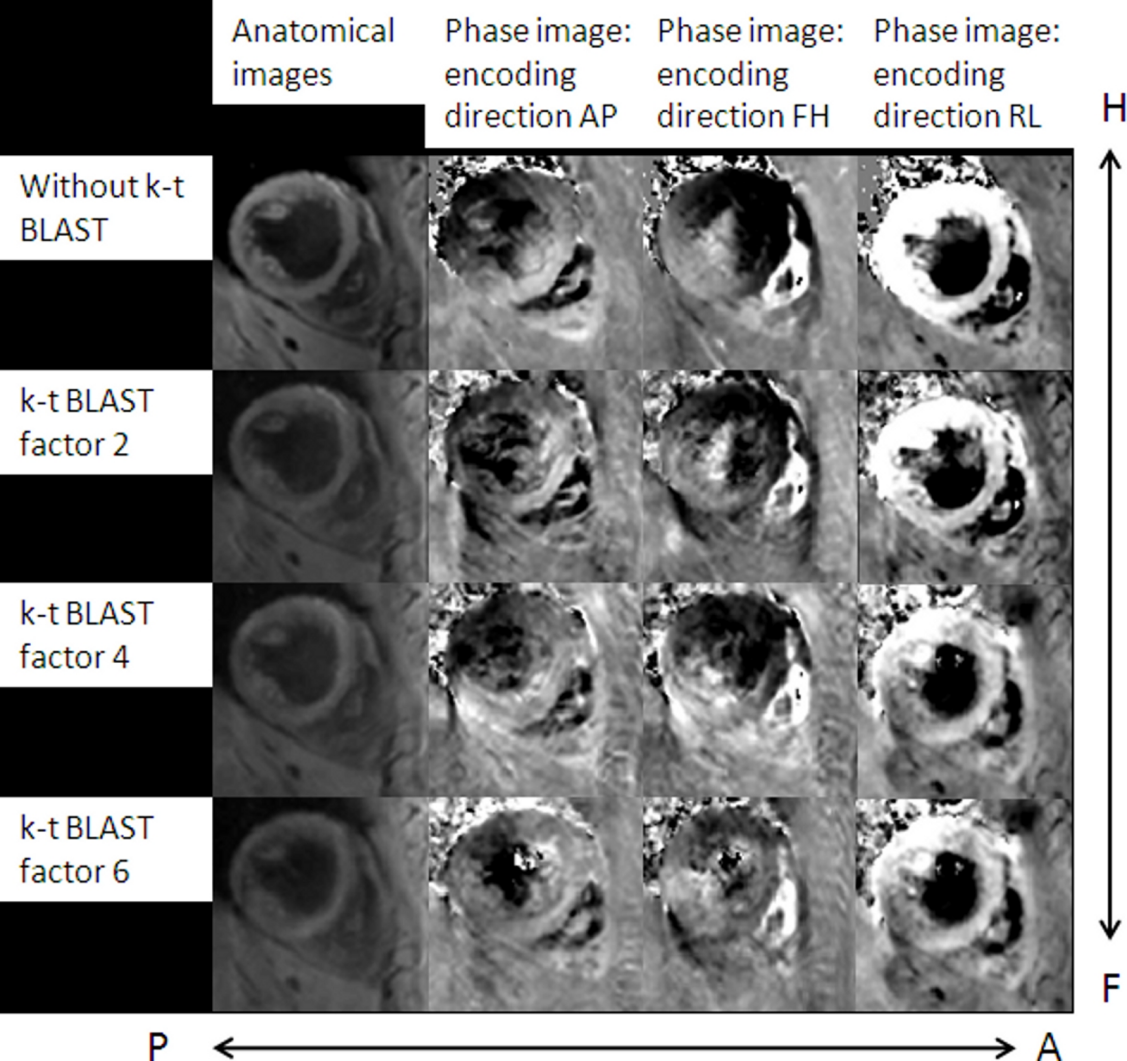
**Anatomical and phase contrast images with and without k-t BLAST**. Anatomical (left) images of the myocardium and respective velocity encoded phase images in AP, FH and RL directions for the conventional sequence and the k-t BLAST accelerated sequences (acceleration factors 2, 4 and 6, heart phase with maximum longitudinal velocity (approx. 50 ms after the R-Wave)

Figure 1 exemplarily shows anatomical and velocity encoded images of the myocardium acquired without and with k-t BLAST acceleration applying acceleration factors of two, four and six. All data were acquired approximately 50 ms after the R-Wave. For low k-t BLAST acceleration factors, the anatomical and phase encoded images do not show obvious deterioration. With increasing k-t BLAST factors, however, an increasing blurring can be observed in the anatomical images. In the TPM data, a reduction of velocity amplitude with increasing k-t BLAST factors can be noticed.

**Figure 2:**
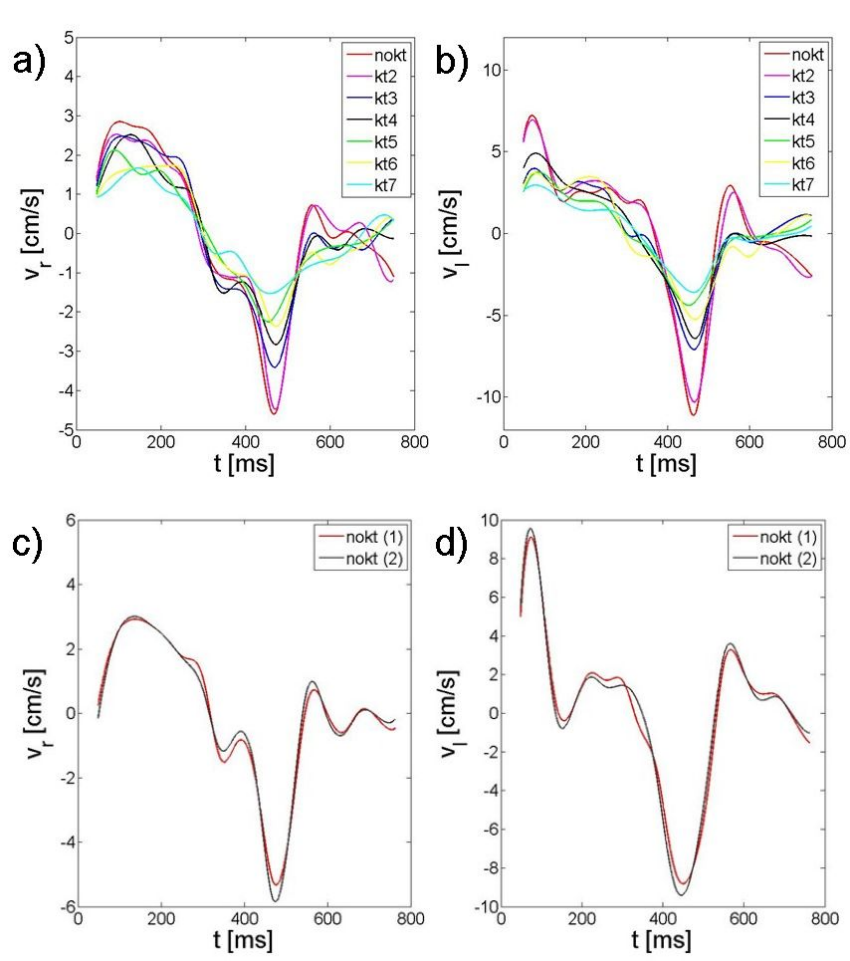
**Velocity curves for different k-t accelerating factors and for the reproducibility study**. In Figure 2 a) and b) the radial and longitudinal velocity curves of a healthy volunteer over the time after the R-peak are displayed for different k-t accelerations (kt-group). In c) and d) two velocity curves without k-t acceleration are displayed (reference group).

Figure 2 shows the radial (a, c) and the longitudinal (b, d) velocity curves over time exemplarily for one volunteer out of the kt-group and one volunteer out of the reference group. The data clearly reveal a decrease of the measured peak velocities with increasing k-t BLAST acceleration factors, which is substantially higher than the reproducibility of the TPM-technique.

The PF analysis provides more quantitative information of the amplitudinal decrease of the velocity curves. Table 2 provides the peak factors PF_r _and PF_l _for the different k-t factors for the radial and longitudinal velocity of the myocardium. PF_r _and PF_l _are both very sensitive to k-t acceleration. A deviation of less than 10% from the expected value of one can only be obtained for a k-t factor of two. Even for this k-t factor, the standard deviation of PF_r _is doubled compared to the reproducibility data, whereas the standard deviation of PF_l _is in the same range. For all investigated k-t BLAST factors, there is a significant (p-value ≤ 0.05) underestimation of the peak-factor.

**Table 2:** Mean radial and longitudinal peak-factors PF_r _and PF_l _and their standard deviations

k-t BLAST factor	PF_r_	σ PF_r_	PF_l_	σ PF_l_
no	1.01	0.06	1.03	0.08
2	0.93	0.13	0.90	0.09
3	0.74	0.13	0.61	0.10
4	0.86	0.12	0.67	0.09
5	0.64	0.10	0.50	0.09
6	0.61	0.14	0.46	0.10
7	0.50	0.12	0.36	0.08

The results of the Bland-Altman analysis of the velocity ranges Δv_r _and Δv_l _for the kt-group and reference group are listed in table 3. Significant increased mean differences Δv_r_Diff and Δv_l_Diff were obtained for increasing acceleration factors.

**Table 3:** Bland-Altman results for the velocity range differences Δv_r_Diff and Δv_l_Diff and their standard deviations

k-t BLAST factor	Δv_r_Diff	σ(Δv_r_Diff)	Δv_l_Diff	σ(Δv_l_Diff)
no	-0.07	0.52	-0.22	1.15
2	0.57	0.88	1.43	1.26
3	1.96	0.98	5.86	1.84
4	1.06	0.93	5.03	1.57
5	2.72	0.91	7.71	2.38
6	2.88	1.03	8.16	2.11
7	3.69	1.07	9.73	2.58

The mean nRMSD_r _and nRMSD_l _values and their standard deviations are listed in table 4. The values reveal an increasing nRMSD value with increasing k-t acceleration. The small nRMSD values for the repeated no-kt measurements (nRMSD_r_: 0.04 ± 0.02; nRMSD_l_: 0.04 ± 0.02) indicate good reproducibility for the TPM technique. All nRMSD_r _and nRMSD_l _values are significantly higher (p-value ≤ 0.05) than the reproducibility of the technique. However, for a k-t BLAST factor of two and four the nRMSD values can be kept below 10%.

**Table 4:** Normalized mean root mean square deviations nRMSD_r _and nRMSD_l _and their standard deviations

k-t BLAST factor	nRMSD_r_	σ nRMSD_r_	nRMSD_l_	σ nRMSD_l_
no	0.04	0.02	0.04	0.02
2	0.08	0.03	0.06	0.03
3	0.10	0.03	0.11	0.01
4	0.09	0.02	0.10	0.02
5	0.13	0.02	0.13	0.02
6	0.14	0.03	0.14	0.02
7	0.16	0.03	0.16	0.02

The mean radial and longitudinal correlation coefficients c_r _and c_l _and their standard deviations are listed in table 5. A significant decrease (p-value < 0.05) of c_r _and c_l _was obtained with increasing k-t BLAST factors. All correlation coefficients are above 0.8.

**Table 5:** Mean radial and longitudinal correlation coefficients c_r _and c_l _and their standard deviations

k-t BLAST factor	c_r_	σ c_r_	c_l_	σ c_l_
no	0.99	0.01	0.99	0.01
2	0.96	0.03	0.97	0.03
3	0.96	0.03	0.90	0.03
4	0.95	0.02	0.91	0.04
5	0.82	0.04	0.88	0.04
6	0.92	0.05	0.86	0.05
7	0.89	0.05	0.85	0.06

The results of the Bland-Altman analysis for the time to the diastolic peak velocity are shown in table 6. For all acceleration factors R Δt_r,dias _and Δt_l,dias _are below 12 ms. For Δt_r,dias _no significant differences could be obtained between the reproducibility measurement and the k-t accelerated measurements. Only for Δt_l,dias _significant differences were obtained for R equal to four, five and seven.

**Table 6:** Results of the Bland-Altman analysis for Δt_r,dias _and Δt_l,dias _and their standard deviations

Compared techniques	Δt_r,dias _[ms]	σ Δt_r,dias _[ms]	Δt_l,dias _[ms]	σ Δt_l,dias _[ms]
[1] No-kt 1 - No-kt 2	2.22	7.52	0.30	5.87
[2] No-kt - kt2	3.03	9.81	4.21	9.78
[3] No-kt - kt3	3.52	13.19	2.76	10.23
[4] No-kt - kt4	4.12	10.71	7.80	14.58
[5] No-kt - kt5	9.54	14.52	8.54	13.52
[6] No-kt - kt6	1.99	13.66	3.71	9.06
[7] No-kt - kt7	8.31	23.97	11.30	15.69

## Discussion

The application of k-t BLAST to accelerate black-blood TPM imaging for the quantification of the myocardial velocity appears feasible. With increasing k-t accelerating factors R an increasing deterioration of the velocity curve and a decrease of the peak velocity is observed. This effect can likely be explained by the inherent slight temporal smoothing of the k-t BLAST algorithm [32,40,42]. With R = 2, the impact on the velocity can be kept very small (≤ 10% deviation from the no-kt measurement for the peak-factors, normalized RMSDs and correlation coefficient and less than 5 ms temporal deviation between the time to the diastolic peak). Nevertheless, the standard deviation of the radial peak-factor was doubled, which might have implications for clinical studies, where the overlap between velocity ranges of healthy volunteers and patients might be increased. Higher acceleration factors show substantial degradation of the peak factors and motion pattern. There is only minimal influence of k-t BLAST on the time to the minimum, indicating the applicability of k-t BLAST for the assessment of temporal behavior.

A similar reduction of the peak velocities was observed in prior work published on the application of k-t BLAST to quantitative flow measurements [32,40,42]. However, in this study a substantial reduction was also observed at lower acceleration factors, which might be attributed to the more complex motion pattern in the case of the myocardium.

In this study, the number of acquired cardiac phases was a multiple integer of the k-t BLAST factor to ensure same undersampling factors in all regions of k-space. Depending on the heart rate and the specific acceleration factor, this might introduce worse temporal sampling, which can also cause lower peak factors [11]. Further improvement may be obtained by an application specific adaptation of the number of cardiac phases. Since only a k-t BLAST factor of two appears reasonable for quantification in tissue phase mapping, choosing multiple integers of the acceleration factor results in the loss of one heart phase at maximum.

Since the possible acceleration by k-t BLAST appears limited to 2, its combination with other acceleration techniques must be considered for further increasing imaging speed. Most promising here may be the combination with parallel imaging techniques (k-t SENSE, t-Grappa [31,49]) or the combination with view sharing.

## Conclusions

In summary, a k-t BLAST factor of two can be applied with statistically significant but not substantial loss of motion information of the myocardium, enabling a 45% decrease in scan duration or a 1.8 fold increase in volume coverage. Higher accelerating factors show substantial degradation of the motion pattern and should therefore be avoided.

The k-t BLAST sequence has the potential to enable 3D tissue phase mapped myocardial imaging with reasonable image acquisition times. The possible combination with parallel imaging techniques offers a way to further reduce the overall scan time.

## Abbreviations

CRT: Cardiac Resynchronization Therapy; DANTE: Delay alternating with nutation for tailored excitation; DENSE: Displacement encoding with stimulated echoes; FOV: Field-of-View; GRAPPA: Generalized autocalibrating partially parallel acquisitions; HARP: Harmonic phase approach; nRMSD: Normalized root mean square deviation; PF: Peak factor; R: k-t Blast acceleration factor; SAR: Specific absorption rate; SENC: Strain-encoding; SENSE: Sensitivity encoding; TPM: Tissue Phase Mapping; UNFOLD: Unaliasing by Fourier-encoding the overlaps using temporal dimension

## Competing interests

VR and AL have a research grant with Philips Healthcare. PE is employed by Philips Healthcare. RM is employed by Philips Research.

## Authors' contributions

AL developed the sequence protocol, performed the data acquisition, the analysis and interpretation of data and drafted the manuscript. AB was involved in developing the sequence and in interpreting the data. PE was involved in the analysis of the data. RM and GUN were involved in the interpretation of the data. VR and AB made substantial contributions to conception and design and revised the manuscript critically for important intellectual content. All authors have given final approval of the version to be published.
